# Activating Interneurons in Local Inhibitory Circuits by High-Frequency Stimulations at the Efferent Fibers of Pyramidal Neurons in Rat Hippocampal CA1 Region

**DOI:** 10.3390/brainsci12101350

**Published:** 2022-10-05

**Authors:** Xiangyu Ye, Zhouyan Feng, Zhaoxiang Wang, Lvpiao Zheng, Yue Yuan, Yifan Hu, Yipeng Xu

**Affiliations:** 1 Key Laboratory for Biomedical Engineering of Ministry of Education, College of Biomedical Engineering and Instrument Science, Zhejiang University, Hangzhou 310027, China; 2 Research Center for Human-Machine Augmented Intelligence, Research Institute of Artificial Intelligence, Zhejiang Lab, Hangzhou 311121, China

**Keywords:** high-frequency stimulation, interneuron, unit spike, population spike, pulse intensity, feedback inhibitory circuit

## Abstract

Stimulation-induced inhibition is one of the important effects of high-frequency stimulation (HFS) utilized by the therapy of deep brain stimulation (DBS) to treat certain neurological diseases such as epilepsy. In order to explore the stimulation sites to induce inhibition, this study investigated the activation effect of HFS of efferent fibers on the local inhibitory interneurons (IN). Antidromic HFS (A-HFS) of 100 Hz pulses was applied for 2 min at the efferent fibers—the alveus (i.e., the axons of pyramidal neurons) in the hippocampal CA1 region of anesthetized rats. Single unit spikes of INs in local feedback inhibitory circuits, as well as antidromically-evoked population spikes (APS) of pyramidal neurons, were recorded simultaneously in the CA1 region upstream of the stimulation site. Results showed that during the late 60 s of A-HFS, with a substantial suppression in APS amplitudes, the mean firing rate of INs was still significantly greater than the baseline level even when the A-HFS was applied with a weak pulse intensity of 0.08 ± 0.05 mA (9 rats). With a strong pulse intensity of 0.33 ± 0.08 mA (10 rats), the mean firing rate of INs was able to keep at a high level till the end of A-HFS. In addition, the mean latency of IN firing was significantly prolonged during the sustained A-HFS, indicating that alterations had been generated in the pathway to activate INs by the stimulations at efferent fibers. The results suggested that HFS at efferent fibers with various stimulation intensities can modulate the firing of local inhibitory neurons. The finding provides new clues for selecting stimulation sites to enhance inhibition in neural circuits by DBS.

## 1. Introduction

Deep brain stimulation (DBS) commonly utilizes high-frequency stimulation (HFS) of electrical pulses to treat neurological disorders, such as Parkinson’s disease and epilepsy [1,2,3]. However, the mechanisms of HFS are inconclusive yet, which limits the development and extension of DBS therapy. DBS has shown an effect on functional lesions similar to that of ablative therapies. The effect can be generated by an HFS-induced depolarization block to prevent the propagation of pathological signals and/or by an HFS-induced correction of unbalance in neuronal excitability [4,5]. For instance, enhancing inhibition in neuronal circuits has been considered one of the important mechanisms of HFS, especially for controlling the over-excitation observable in certain neurological disorders such as epilepsy [5,6]. Previous studies have shown that HFS can enhance inhibition by directly stimulating the inhibitory neurons or the presynaptic segments of inhibitory synapses [7,8]. However, to activate inhibitory neurons effectively by a micro-size stimulation electrode, more stimulation sites are needed to be explored to meet various treatment requirements. In the present study, we explored a new stimulation site to enhance the firing of inhibitory neurons.

**Figure 1:**
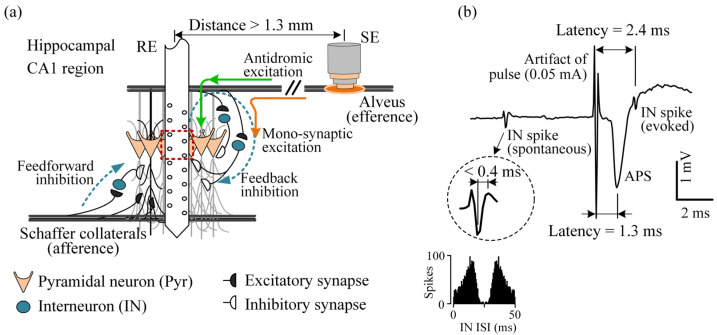
Responses of pyramidal neurons (Pyr) and interneurons (IN) to the antidromic stimulation at the alveus in the rat hippocampal CA1 region. (**a**) Schematic diagram of the local inhibitory neural circuits and the electrode locations. The Pyrs accept inhibitory input from both feedforward (denoted by the blue arrow on the left) and feedback (denoted by the blue arrow on the right) inhibitory circuits. The INs in the inhibitory circuits are activated by excitatory synapses (solid semicircles) and then act on the somata or dendrites of Pyrs via inhibitory synapses (hollow semicircles). The excitation generated by an electrical pulse applied at the alveus (axons of the Pyrs) by the stimulation electrode (SE) can propagate antidromically along the axons and then activate the somata of the Pyrs (green curve with arrows). Simultaneously, the excitation may activate the INs in the feedback inhibitory circuits via monosynaptic transmissions (orange curve with arrow). The red dashed box denotes the channels on the recording electrode (RE) located in the pyramidal layer of CA1 region. (**b**) Antidromically-evoked population spike (APS) and a unit spike of an IN evoked by a single-pulse stimulation at the alveus. The expanded waveform of a typical IN spike and the histogram of inter-spike-intervals (ISI) of IN spikes in the baseline state were used to identify the IN firing.

The hippocampus is one of the common brain regions of epileptic foci and is becoming a potential DBS target for treating refractory epilepsy [3,9]. However, stimulation sites in the hippocampal region are not determined yet, waiting for more experimental investigations to provide clues. In the hippocampus, the balance of excitability of principal neurons is adjusted by the activity of inhibitory interneurons in the two types of local neural circuits: feedforward and feedback inhibitory circuits [10]. The interneurons in the feedforward circuits receive activations from the afferent pathway/axons that simultaneously innervate principal neurons and interneurons. The excited interneurons then inhibit the principal neurons through their inhibitory synapses (Figure 1a). Meanwhile, the interneurons in the feedback circuits receive activations from axonal branches of neighboring principal neurons and then return to inhibit the principal neurons. If the application of HFS used by DBS could activate the interneurons in the local circuits, the principal neurons would be inhibited. In the present study, we investigated whether HFS at the efferent fiber/axons of the hippocampus could modulate the firing of local interneurons.

Axons are more prone to be excited by the narrow pulses of HFS than the other neuronal elements, such as somata and dendrites [11,12,13]. The action potential induced by a pulse on the axonal membrane can simultaneously propagate along the axon in two directions: orthodromic propagation to the axonal terminals and antidromic propagation to the neuronal soma [9]. Studies have shown that through orthodromic propagations, HFS at afferent axons can increase the firing of the postsynaptic neurons (including the interneurons of feedforward circuits) in the downstream area of the stimulation site via synaptic transmissions at axonal terminals [14,15,16]. However, it is not clear yet whether the HFS-induced excitation at efferent axons can activate the interneurons of local feedback circuits in the upstream area of the stimulation site through antidromic propagations (see Figure 1a).

To address the question, we performed experiments on anesthetized rats in vivo and applied antidromic HFS (A-HFS) at the efferent pathway of the hippocampal region, i.e., the alveus formed by the axons of principal neurons—the pyramidal neurons of hippocampal CA1 region [17]. In order to quantify the modulation effects of A-HFS on the interneurons in the feedback inhibitory circuits, single unit spikes of interneurons as well as antidromically-evoked population spikes (APS) of pyramidal neurons were recorded simultaneously in the CA1 region upstream of the stimulation site. The results of the study can provide new information for selecting stimulation targets and for understanding more mechanisms of DBS therapy.

## 2. Materials and Methods

### 2.1. Animal Surgery and Electrode Implantation

The animal experiment protocol was approved by the Institutional Animal Care and Ethics Committee, Zhejiang University. Nineteen adult male Sprague-Dawley rats (357 ± 48 g) were anesthetized by an intraperitoneal injection of urethane (~1.25 g/kg) and fixed in a stereotaxic apparatus (Stoelting Co., Wood Dale, IL, USA). Similar to our previous report [18], the skin over the nasal bone was cut open. Two stainless steel screws were fixed apart on the exposed nasal bone to serve as the reference and ground electrodes for neural electrical signal recordings. The skin over the skull was cut open along the sagittal midline, and a part of the skull over the left brain was removed. Then, the recording electrode (RE) and the stimulation electrode (SE) were inserted into the left hippocampus through the cerebral cortex.

The RE was a 16-channel electrode array (A1x16-Poly2-5mm-50-177, NeuroNexus Technologies Inc., Ann Arbor, MI, USA) and was implanted vertically into the hippocampal CA1 region (AP −3.5 mm, ML 2.7 mm, DV ~2.5 mm). The SE was a bipolar concentric electrode (CBCSG75, FHC Inc., Bowdoin, ME, USA; inner pole: platinum-iridium, diameter 75 µm; outer pole: stainless steel, diameter 250 µm) and was implanted at the alveus (AP −4.8 mm, ML 2.7 mm, DV ~2.3 mm) to antidromically activate the CA1 pyramidal neurons directly and activate the interneurons (IN) in the local circuits via monosynaptic transmissions (Figure 1a). The correct placement of the electrodes was determined by the unique waveforms of evoked potentials appearing along the recording channels of the RE array located across the laminar structures of CA1 region [19]. The unit spikes of spontaneous neuronal firing appearing intensively on the channels within or near the pyramidal layer of CA1 region (i.e., the soma layer denoted by the red dashed box in Figure 1a) were also used to assist the RE placement.

### 2.2. Stimulating and Recording

The stimulation pulses were biphasic current pulses with a first cathodic phase followed by a second anodic phase. Each phase was a rectangle with a width of 100 µs. The pulses were generated by a programmable stimulator (Model 3800, A-M Systems Inc., Sequim, WA, USA) and then delivered to the SE through a stimulus isolator (Model 3820, A-M Systems Inc., Sequim, WA, USA).

A-HFS sequences were applied with a duration of 2 min and a pulse frequency of 100 Hz in the DBS frequency range of 50–200 Hz [2,3,20]. The pulse intensity was divided into weak and strong groups. The mean intensity of the weak group was 0.08 ± 0.05 mA (9 rats)—the minimum current that was able to reliably induce a unit spike of the identified IN in six trials of single-pulse stimulation with an interval longer than 10 s. The mean intensity of the strong group was 0.33 ± 0.08 mA (10 rats), which was able to evoke an APS with an amplitude of ~3/4 maximum.

The neural electrical signals collected by the RE array were amplified 100-fold in a frequency range of 0.3–5000 Hz by a 16-channel amplifier with a headstage (Model 3600, A-M Systems Inc., Sequim, WA, USA). The amplified signals were then amplified further and sampled by a PowerLab data acquisition system (Model PL3516, ADInstruments Inc., Bella Vista, NSW, Australia) with a sampling rate of 20 kHz per channel.

### 2.3. Detection and Classification of Unit Spikes

In order to investigate the modulation effect of A-HFS on the INs of feedback circuits, unit spikes of specific INs were collected. First, the amplitude of the unit spikes was greater than 100 μV at least at one of the RE channels to ensure reliable identifications of the unit spikes with interference of evoked APS during A-HFS periods. Second, the IN should be innervated by the alveus stimulation, which was indicated by a clear phase-locking relationship between its firing and the A-HFS pulses. Third, the evoked IN spikes should have a latency conforming to a monosynaptic transmission to show a putative location of the IN in the feedback circuits (see Figure 1a). Due to the sparse distribution of INs in the CA1 region, a careful adjustment of the RE position was performed to obtain the unit spikes of required IN during A-HFS in each rat experiment. 

After the signals with IN spikes were recorded, the recording in the channel with the greatest amplitude of IN spikes, together with the recordings of three adjacent channels, were used to perform the following process to detect the spikes. The process was a semi-automatic method based on the detection of steep falling phase of unit spikes using a moving window. First, the width and amplitude of the falling phase of putative IN spikes were pre-estimated manually in the channel with the greatest spike amplitude. Accordingly, the lower (50–200 μV) and the upper (200–700 μV) limits of spike amplitudes were set for the detection window together with a width of the window set at 0.2 or 0.4 ms in a custom-made MATLAB software (MathWorks Inc., Natick, MA, USA). Then, the software was run to automatically detect unit spikes by moving the detection window along the original recordings without overlap. Once the maximum potential difference in the window was within the range defined by the lower and upper limits, a putative unit spike was captured. After the unit spikes in all of the four channels were detected in turn, the detected spikes were pooled, and the duplicate detections were removed.

Next, the waveforms of the detected unit spikes were extracted and used for the following process of spike sorting, similar to the previous reports [15]. First, eight feature vectors were calculated, including the first principal component and the peak-to-peak amplitude of the spike waveforms from the four channel recordings. Then, the spike sorting was performed by using the open-source software SpikeSort 3D (Neuralynx Inc., Bozeman, MT, USA) with the feature vectors. According to the sorting results presented on the graphical interface of the software, appropriate manual adjustments were made to ensure sorting correctness. Finally, unit spikes of INs were determined according to the spike waveform with a rising phase shorter than 0.4 ms (Figure 1b), as well as the smooth histogram of inter-spike-interval (ISI) obtained in baseline recording [21]. The amplitude of IN spikes in each rat experiment was defined as the mean amplitude measured on the accumulated waveforms of all the obtained spikes both at baseline and during A-HFS.

### 2.4. Data Analysis

The APS evoked by a stimulation pulse at the axons of pyramidal neurons is formed by the integration of synchronous firing of action potentials generated in the somata of a population of neurons. The amplitude of APS can be used to evaluate the number of activated neurons [22], and the latency of APS, the time required to activate the neuronal somata following the application of pulse at their axons, can reflect changes in the activation pathway. Thus, based on the recording signal from the channel with the maximum APS amplitude located in the pyramidal layer of CA1 region, we calculated the APS amplitude (i.e., the potential difference of the falling phase of APS waveform) and the APS latency (i.e., the time difference between the preceding A-HFS pulse and the negative peak of APS waveform). Because the evoked APS was suppressed substantially by A-HFS, to improve the signal-to-noise ratio, the two APS indexes were measured as a mean value per second during A-HFS, except for the first APS evoked by the first pulse of A-HFS. That is, the 100 waveforms of evoked APS per second were accumulated before the indexes were measured.

In order to show the phase-locking relationship between an IN spike and the preceding pulse of A-HFS, the post-stimulus time (PST) of each spike in the 10 ms inter-pulse interval of A-HFS was calculated. The PST data were then used to establish post-stimulus time histograms (PSTH) with a bin size of 0.5 ms [23]. In order to quantify the phase-locking of IN spikes, the interquartile range of PST (IQR_PST_) was calculated. A smaller IQR_PST_ indicated a stronger phase-locking between the IN firing and the A-HFS pulses.

Statistical data were represented as mean ± standard deviation. One-way ANOVA with post hoc Bonferroni tests and *t*-test were used to judge the statistical significance of differences among data groups.

## 3. Results

### 3.1. Distinguish the Unit Spikes of Interneuron in Feedback Inhibitory Circuits

A single pulse stimulation applied at the alveus can activate a bundle of axons and generate action potentials that propagate antidromically to the somata of pyramidal neurons, thereby generating an APS around the somata (Figure 1). As recorded in a typical experiment (Figure 1b), immediately following the evoked APS by a pulse of 0.05 mA, a unit spike of IN appeared with a latency (2.4 ms) longer than the latency of APS (1.3 ms). The longer latency of IN spike conformed to a delay caused by a monosynaptic transmission [24], indicating that the IN was in an inhibitory feedback circuit (Figure 1a).

In the total 19 rat experiments, nineteen INs were obtained (one per experiment), all of which were innervated by the alveus stimulation with a mean latency (2.7 ± 0.45 ms) in the range of monosynaptic transmissions. To investigate the IN firing responding to A-HFS at the alveus and the effect of stimulation intensities, A-HFS was performed in two rat groups: the weak-intensity group (9 rats) and the strong-intensity group (10 rats). The results are presented below.

### 3.2. Firing of Interneurons in the Feedback Circuits during Weak A-HFS

**Figure 2:**
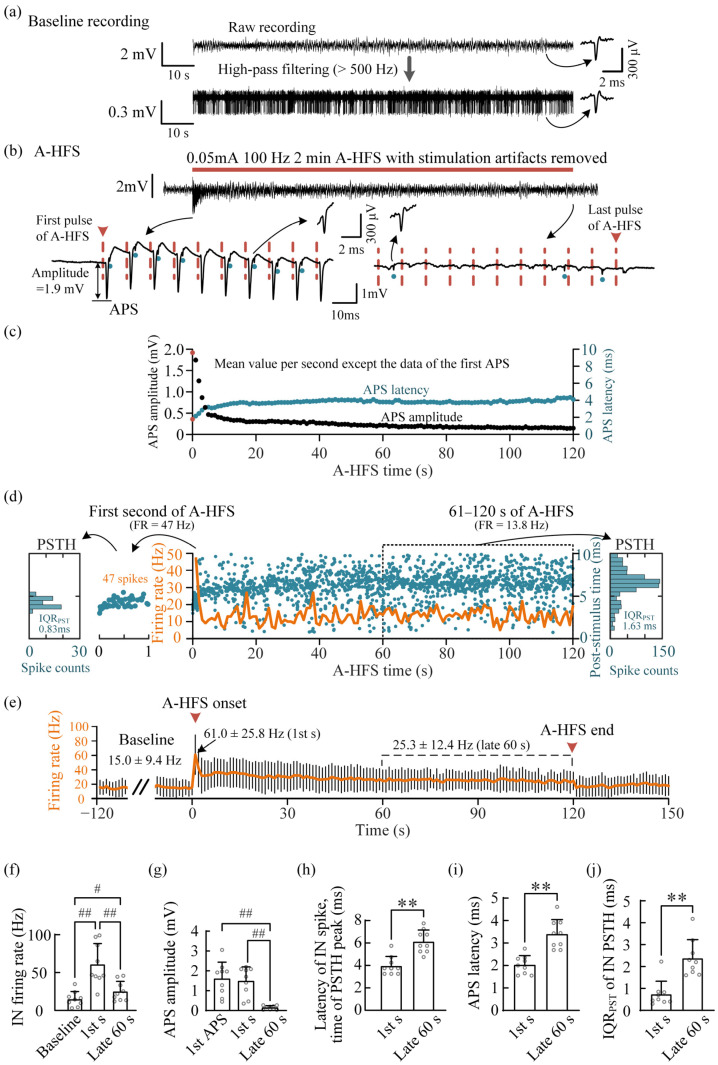
Modulating the firing of IN in the feedback circuit by weak A-HFS at the efferent axons of the hippocampal CA1 region. (**a**) Baseline recording and its high-pass filtered signal with obvious unit spikes of an IN. (**b**) Evoked APS and IN spikes during A-HFS. The red horizontal bar denotes the A-HFS period and the dashed vertical lines denote the stimulation pulses. The blue dots mark the unit spikes of IN. (**c**) Scatter plots of the amplitudes and the latencies of evoked APSs during A-HFS. Except for the data of the first APS (red dots), the other data were the mean values per second. (**d**) Two-dimensional raster plot of the unit spikes of IN firing during A-HFS (blue dots) together with its firing rate (FR) per second (orange curve). The post-stimulus time histogram (PSTH) plots of the 1st second and of the late 60 s (61–120 s) of A-HFS are shown on the left and right, respectively. (**e**) Mean firing rates per second of nine INs before, during and after the weak A-HFS. The black lines denote the standard deviations. The mean firing rates in the baseline, in the 1st second and in the late 60 s of A-HFS are written. (**f**,**g**) Comparisons of mean firing rates of IN (**f**) and mean APS amplitudes (**g**) in the baseline, in the 1st second and in the late 60 s of A-HFS. # *p* < 0.05, ## *p* < 0.01, repeated measurements one-way ANOVA with post hoc Bonferroni tests, 9 rats. (**h**,**i**) Comparison of the mean times of IN PSTH peaks (**h**) and mean APS latencies (**i**) between the 1st second and the late 60 s of A-HFS. (**j**) Comparison of the mean interquartile range of post-stimulus time (IQR_PST_) of IN PSTH between the 1st second and the late 60 s of A-HFS. In (**h**–**j**), ** *p* < 0.01, paired *t-*test, 9 rats.

One of the experimental recordings is shown in Figure 2a–d. In the baseline recording in the pyramidal layer of the CA1 region, the spontaneous firing of an IN was clear, especially in the high-pass filtered signal with a cut-off frequency of 500 Hz to remove the local field potentials in the low-frequency band. The mean firing rate of the IN in the example was 9.4 Hz during the 2-min baseline recording. To investigate the effect of A-HFS on the IN, a 2-min sequence of 100 Hz A-HFS was applied at the alveus with a weak intensity of 0.05 mA to avoid evoking large APSs to interfere with the detection of IN firing (Figure 2b). At the beginning of A-HFS, each pulse induced an APS with an amplitude of ~1.9 mV. The observed IN fired immediately following APS with a high coupling rate of ~80% (Figure 2b bottom left). As the A-HFS proceeded, along with a decrease in APS amplitudes, the coupling rate of IN firing decreased to <13.8% in the late 60 s of A-HFS (Figure 2b bottom right, Figure 2c,d).

The mean firing rate (FR) of the IN at the first second of A-HFS was 47 Hz. The firing concentrated within a narrow range of 3.0–5.5 ms in the 10 ms inter-pulse-interval with a small IQR_PST_ of 0.83 ms, indicating a strong phase-locking of the firing to the pulses (Figure 2d left). However, in the late 60 s of A-HFS, the mean firing rate of IN decreased to 13.8 Hz, and the IQR_PST_ increased to 1.63 ms. Though the firing rate was still greater than the baseline firing rate (9.4 Hz), some of the firings were not phase-locked with the A-HFS pulses. With the gradual increase in the APS latency (the blue dots in Figure 2c), the latency of stimulation-induced IN spikes also increased from 3.00 ms at the onset of A-HFS to a mean value of 6.75 ms in the late 60 s (i.e., the peak time of PSTH shown on the right of Figure 2d).

For all the experiments of nine rats with A-HFS of weak-intensity pulses (0.08 ± 0.05 mA), the statistical data showed that the firing rate of IN in the late 60 s of A-HFS was significantly lower than that in the first second of A-HFS but greater than that in the baseline (Figure 2e,f). Meanwhile, the mean APS amplitude in the late 60 s of A-HFS was significantly smaller than both in the first second of A-HFS and in baseline (i.e., the first APS evoked by the first pulse of A-HFS) (Figure 2g). In addition, the latency of stimulation-induced IN spikes in the late 60 s of A-HFS was significantly longer than that in the first second (Figure 2h). Furthermore, the APS latency in the late 60 s of A-HFS was significantly longer than that in the first second (Figure 2i). Moreover, the IQR_PST_ of IN PSTH in the late 60 s was significantly greater than that in the first second (Figure 2j). These results suggested a progressive decrease in the activation effect of A-HFS on the firing of IN during the weak A-HFS.

### 3.3. Comparing the Modulation Effects on the Firing of Interneurons by A-HFS with Strong- and Weak-Intensity Pulses

**Figure 3:**
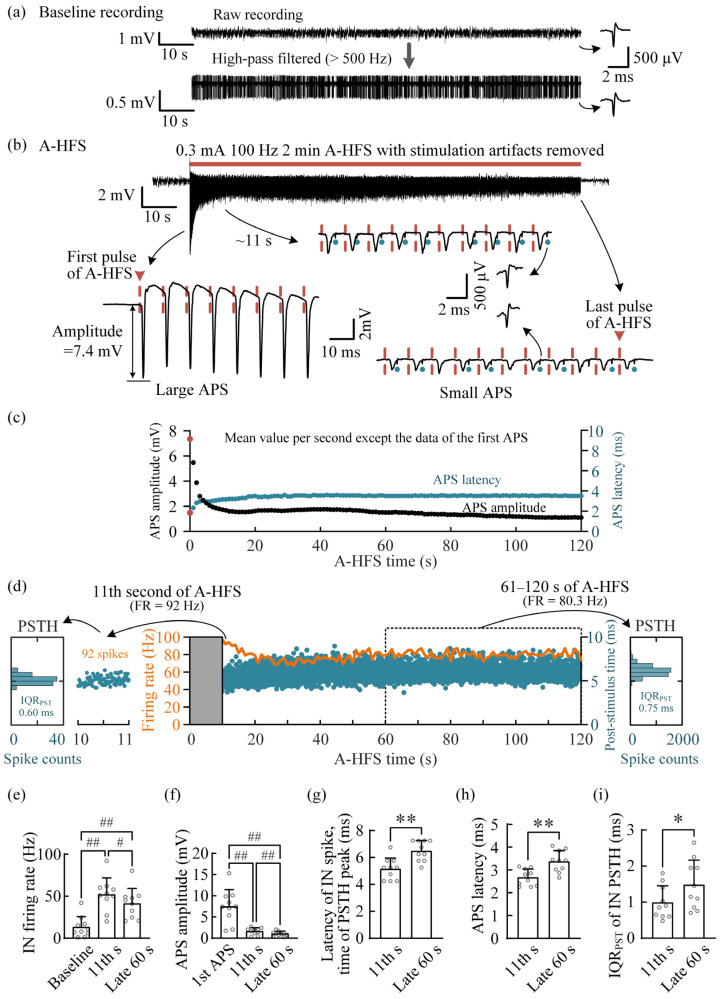
Modulating the firing of IN in the feedback circuit by strong A-HFS. (**a**) Baseline recording and its high-pass filtered signal with obvious unit spikes of an IN. (**b**) Evoked APS and IN spikes during A-HFS. (**c**) Scatter plots of the amplitudes and the latencies of evoked APSs during A-HFS. (**d**) Two-dimensional raster plot of the unit spikes of IN firing during A-HFS (blue dots) together with its firing rate per second (orange curve). The PSTH plots of the 11th second and late 60 s (61–120 s) of A-HFS are shown on the left and right, respectively. (**e**,**f**) Comparisons of mean firing rates of IN (**e**) and mean APS amplitudes (**f**) in the baseline, in the 11th second and in the late 60 s of A-HFS. # *p* < 0.05, ## *p* < 0.01, repeated measurements one-way ANOVA with post hoc Bonferroni tests, 10 rats. (**g**,**h**) Comparison of the mean times of IN PSTH peaks (**g**) and mean APS latencies (**h**) between the 11th second and the late 60 s of A-HFS. (**i**) Comparison of the mean IQR_PST_ of IN PSTH between the 11th second and the late 60 s of A-HFS. In (**g**–**i**), * *p* < 0.05, ** *p* < 0.01, paired *t-*test, 10 rats.

In order to enhance the effect of A-HFS on IN firing, the pulse intensity was increased to a strong level that was able to induce an APS with ~3/4 maximum amplitude in the baseline. An example of the experiments is shown in Figure 3a–d. With clear IN spikes recorded at baseline (Figure 3a), an A-HFS sequence with strong intensity of 0.3 mA was applied at the alveus (Figure 3b). In the initial period, each pulse induced a large APS with an amplitude > 7 mV. Distinguishing the IN firing in this period was difficult due to the large APSs. After ~11 s stimulation of the A-HFS, with the suppression of APS (Figure 3b,c), the stimulation-induced unit spikes of IN clearly appeared following the APS waveforms (denoted by the blue dots in the insets of Figure 3b). The firing rate of the IN at the 11th second of A-HFS was 92 Hz (Figure 3d) with an IQR_PST_ of 0.60 ms in the PSTH. The firing rate of IN kept at a high level till the end of A-HFS. In the late 60 s of A-HFS, the mean firing rate was 80.3 Hz with a still small IQR_PST_ of 0.75 ms.

The statistical data of all 10 rat experiments showed that the firing rate of IN in the late 60 s of A-HFS was significantly lower than that in the 11th second of A-HFS but greater than that in the baseline (Figure 3e). Meanwhile, the mean APS amplitude in the late 60 s of A-HFS was significantly smaller than both in the 11th second of A-HFS and in baseline (Figure 3f). In addition, the latency of stimulation-induced IN spikes in the late 60 s of A-HFS was significantly longer than that in the 11th second (Figure 3g), and the APS latency in the late 60 s of A-HFS was significantly longer than that in the 11th second (Figure 3h), indicating a positive correlation between the two types of latency. Moreover, the IQR_PST_ of IN PSTH in the late 60 s was significantly greater than that in the 11th second, but the mean increment was only 0.49 ± 0.57 ms (Figure 3i).

**Figure 4:**
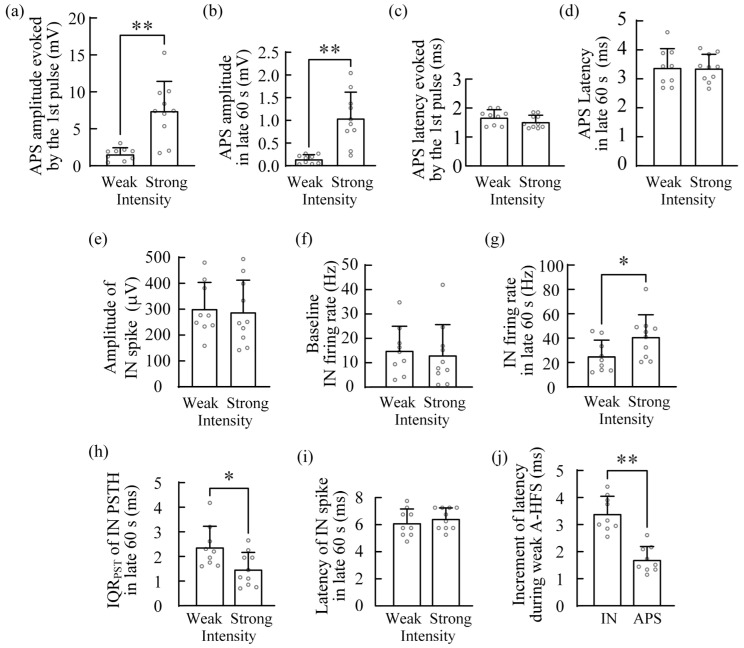
Comparisons of the data between the two A-HFS groups with weak- and strong-intensity pulses. (**a**–**d**) Comparisons of the mean initial APS amplitudes (**a**), the mean APS amplitudes in the late 60 s of A-HFS (**b**), the mean initial APS latencies (**c**) and the mean APS latencies in the late 60 s of A-HFS (**d**). (**e**–**g**) Comparisons of the amplitudes of IN spikes (**e**), the baseline IN firing rates (**f**) and the IN firing rates in the late 60 s of A-HFS (**g**). (**h**) Comparison of IQR_PST_ of IN firing in the late 60 s of A-HFS. (**i**) Comparison of the latency of IN spikes in the late 60 s of A-HFS. (**j**) Comparison of the latency increments (i.e., for IN spike: the peak time of PSTH in the late 60 s minus the latency following the 1st pulse; for APS: mean latency in the late 60 s minus the latency following the 1st pulse.) between the IN spikes and the APS caused by weak A-HFS. * *p* < 0.05, ** *p* < 0.01, (**a**–**i**) unpaired *t*-test, (**j**) paired *t*-test, weak group: *n* = 9 rats, strong group: *n* = 10 rats.

The comparisons between the two groups of experiments with different intensities showed that the mean APS amplitude evoked by the first pulse of A-HFS (representing the baseline value) with the strong intensity (0.33 ± 0.08 mA) was significantly greater than the corresponding value with the weak-intensity (0.08 ± 0.05 mA) (Figure 4a). The significant difference between the APS amplitudes maintained to the late 60 s of A-HFS (Figure 4b) with substantial decreases in the APS amplitudes. It indicated a significant difference in the number of activated pyramidal neurons by the different intensities. However, the mean APS latencies of the two groups were not significantly different, although the latencies increased about twice from the initial value to the mean value in the late 60 s of A-HFS (Figure 4c,d), indicating no significant difference in the activation pathway of the two groups.

For the IN firing, with similar spike amplitudes (Figure 4e) and with similar baseline excitability indicated by the similar baseline firing rates (Figure 4f), in the late 60 s of A-HFS, the mean firing rate of INs of the strong group was significantly greater than that of the weak group (Figure 4g). In addition, the mean IQR_PST_ of the strong group was significantly smaller than that of the weak group (Figure 4h), indicating a stronger phase-locking of the strong group. However, the mean latencies of IN spikes of the two groups were not significantly different (Figure 4i), indicating a similar activation pathway of the INs.

In addition, in the weak group, the latency of IN spike increased significantly from the initial value of 2.73 ± 0.58 ms evoked by the first pulse to 6.14 ± 0.97 ms in the late 60 s of A-HFS. The latency increment of 3.41 ± 0.61 ms was significantly greater than the increment of APS latency of 1.71 ± 0.45 ms (Figure 4j), indicating that the delay generated by A-HFS in the activation pathway of INs was significantly greater than that in the antidromic activation pathway of pyramidal neurons.

Taken together, because the strong A-HFS was able to activate axons of a larger population of pyramidal neurons indicated by the greater APS, the IN firing increased more with a stronger phase-locking by the strong A-HFS than the weak A-HFS. However, the latencies of both APS and IN firing were similar for the two groups of A-HFS, while the latency increment was greater in IN firing than in APS.

## 4. Discussion

Being different from inducing inhibition by directly stimulating inhibitory neurons in previous studies, our present study showed that in the rat hippocampal CA1 region, 100 Hz A-HFS at the efferent fibers of principal neurons was able to increase the firing of INs in local inhibitory circuits upstream of the stimulation site. Nevertheless, the IN firing was attenuated substantially in the late period of weak A-HFS, while the IN firing was able to keep at a high rate till the end of strong A-HFS. The advantages of the experiment method, as well as the possible underlying mechanisms and implications, are analyzed below.

### 4.1. Advantages of the Experiment Method

To the best of our knowledge, this is the first study investigating the modulation of the local hippocampal interneurons by A-HFS at the efferent fibers of principal neurons in vivo. By utilizing the difference between the latency of monosynaptic activation of IN and the latency of direct activation of pyramidal neurons, the unit spikes of INs were able to be distinguished reliably following APS (Figure 2b and Figure 3b), even though the amplitude of unit spikes of IN was far smaller than the amplitude of population spikes, i.e., APS. To ensure the correct detection of IN spikes, all the nineteen INs used here had a spike amplitude >100 μV, and the mean spike amplitude was ~300 μV (Figure 4e).

The unit spikes of IN obtained here were activated by the stimulation pulses applied at the alveus with a baseline latency (2.7 ± 0.45 ms) in the range of monosynaptic activation [24]. According to the structures of the local inhibitory circuits in the hippocampal CA1 region [10,25], the detected INs were putatively in the feedback inhibitory circuits (Figure 1a). Because the recording site was >1.3 mm upstream of the stimulation site, the excitation induced at the efferent axons by the stimulation should have propagated antidromically along the axons to close to the INs in the recording site before activating the INs through axon branches (see the pathway denoted by the orange curve in Figure 1a). The experiment provides a new method to investigate the antidromic activations of efferent fibers on the local INs rather than the orthodromic activations of afferent fibers, as in previous studies [14,15,16].

Compared with direct stimulations on inhibitory neurons or on presynaptic segments of inhibitory synapses [7,8], the effect of stimulation of a bundle of axonal fibers can spread out through axonal projections to activate neurons in a large range. Thus, even a micro-size electrode may generate a substantial effect, just as the stimulation of the alveus here. The alveus is a compact layer of axonal fibers lying in the ventricle and covering the dorsal side of the hippocampus [17]. Therefore, activating neurons by stimulating the alveus may be highly efficient.

### 4.2. Possible Underlying Mechanisms

The amplitude of APS can indicate the number of axons activated by each pulse of A-HFS. Previous studies have shown that only at the beginning of 100 Hz A-HFS, the axons under the stimulation can follow most of the A-HFS pulses to generate action potentials to antidromically activate the somata of pyramidal neurons. Sustained A-HFS can induce depolarization block and result in the axons only intermittently following a part of the pulses to generate action potentials, thereby suppressing the evoked APS [4,18], as shown in Figure 2c, Figure 3c and Figure 4a,b. Presumably, the axonal block would also decrease the activations propagating to the INs in the feedback inhibitory circuits. Therefore, the firing rate of INs decreased rapidly during the initial period of A-HFS, along with the decrease in APS amplitudes (Figure 2). Nevertheless, during A-HFS with a strong intensity, the firing rate of IN was able to keep at a higher level than that during weak A-HFS (Figure 3 and Figure 4g), indicating that the firing rate of IN can be adjusted by the pulse intensity. This can be explained by the characteristics of IN.

The number of IN in the hippocampal CA1 region is only about 10% of the number of pyramidal neurons [17]. Each IN in inhibitory circuits accepts excitatory inputs from numerous pyramidal neurons. However, activating an IN to fire only needs a simultaneous arrival of a few of the inputs due to the low threshold of IN activation [24]. Therefore, even with weak intensity, the pulses of A-HFS were still able to activate an IN to fire at a coupling rate of ~80% at the beginning of A-HFS (Figure 2b) and at a mean rate as high as 61 ± 26 Hz at the first second of A-HFS (Figure 2f). Similarly, in the late period of strong A-HFS, when the axonal block substantially decreased the activation inputs indicated by the suppression of APS amplitude to ~15% of the initial level (Figure 4a,b), an IN was still able to follow the pulses to fire at a mean rate as high as ~40 Hz (i.e., a coupling rate of ~40%) with a strong phase-locking to the pulses of A-HFS (Figure 4g,h). Presumably, during the late period of A-HFS, the suppressed inputs from intermittently activated axons were still sufficient to activate the INs at a high coupling rate (Figure 3).

In addition, the axonal block can delay the generation and propagation of stimulation-induced neuronal responses [18,26,27]. Therefore, both the latencies of APS and IN firing significantly increased by the sustained A-HFS. For the same activation pathway, the increases in latencies were similar (Figure 4c,d,i). However, the increment of latency in the activation pathway of IN was greater than that in the activation pathway of pyramidal neurons (Figure 4j), indicating that additional delays should have been generated at certain sections of IN pathway apart from the delays at the stimulation site and at the axon segment shared by the two pathways (Figure 1a). Presumably, the additional delay may generate at the turning point where the antidromic propagation of induced activation turns into the axonal branch to reach the IN [28,29,30]. In addition, the invasion of induced activation with a high rate may also slow down the propagation along the axonal branch and at the synaptic transmission of axonal terminals in the pathway reaching the IN [31]. Therefore, the complex structures in the activation pathway of INs may cause a longer delay than in the direct activation pathway of pyramidal neurons.

Taken together, the mechanism of HFS-induced axonal block may cause the attenuated firing and the prolonged latency in IN responses to the HFS at efferent fibers, and the firing rate of INs can be modulated in a wide range by various stimulation intensities.

### 4.3. Implications and Limitations

In the present study, with intact neural circuits in the rat experiments in vivo, the results show important information that efferent fiber stimulations can modulate local inhibitory neurons. It provides clues for the selection of stimulation sites for DBS therapies. Although each axon in the efferent fibers (alveus) is from the sole soma of a pyramidal neuron, the axon can innervate many INs through its numerous branches, and the INs can then return to control pyramidal neurons in a wide range [17]. Therefore, increasing the inhibition of principal neurons by stimulating the efferent fibers to activate INs may be a potential strategy to treat certain diseases such as epilepsy [1,3,32]. The hippocampus is one of the common epileptic foci. Epileptiform activity is apt to appear in the CA3 region, where the excitability of pyramidal neurons is high due to the abundant interconnections among the excitatory pyramidal neurons [17,33,34]. The epileptiform activity of the CA3 region can propagate to the downstream CA1 region and then travel to other brain regions through the efferent fibers, i.e., the alveus [33,34,35,36]. If the application of DBS at efferent fibers could inhibit the CA1 pyramidal neurons, the propagation of epileptiform activity would be stopped and eliminated.

Nevertheless, the detection of IN firing here was performed by using extracellular recordings. Although the in vivo experiments can maintain intact neural circuits, further studies of intracellular recordings may provide more detailed information on IN activity induced by HFS. In addition, further studies are needed to reveal the effects of the HFS-induced IN firing on the activity of the principal neurons. Consider that distinct interneurons may interact with each other [37], and intense activation may even change the inhibitory effect of interneurons into an excitatory effect [38]. The transient dynamics of IN firing in the initial period of A-HFS and the intensity-dependent firing rates during sustained A-HFS may generate complex modulation effects that deserve further investigation. Furthermore, the efficacy of this stimulation mode in controlling pathological states of neuronal also needs further investigations to verify.

## 5. Conclusions

This electrophysiological study in rat hippocampus in vivo showed that HFS at the efferent axons of the principal neurons was able to increase the firing of upstream interneurons in local inhibitory circuits. The finding provides important clues for selecting the stimulation targets for controlling the excitability of principal neurons.

## Data Availability

The data presented in this study are available on request from the corresponding author.
